# Research on neglected tropical diseases in Haiti: a scientometrics analysis, 1923-2022

**DOI:** 10.1590/0102-311XEN020824

**Published:** 2025-02-24

**Authors:** Melicile Glesil, Adjoane Maurício Silva Maciel, Taynara Lais Silva, Anderson Fuentes Ferreira, Aymee Medeiros da Rocha, Gabriela Soledad Márdero García, Evens Emmanuel, Max François Millien, Jorg Heukelbach, Eliana Amorim de Souza, Ana Raquel da Silva Paz, Alberto Novaes Ramos

**Affiliations:** 1 Faculdade de Medicina, Universidade Federal do Ceará, Fortaleza, Brasil.; 2 NHR Brasil, Fortaleza, Brasil.; 3 Secretaria Municipal de Saúde, Russas, Brasil.; 4 Universidade Quisqueya, Port-au-Prince, Haiti.; 5 Instituto Multidisciplinar em Saúde, Universidade Federal da Bahia, Vitória da Conquista, Brasil.; 6 Faculdade de Farmácia, Odontologia e Enfermagem, Universidade Federal do Ceará, Fortaleza, Brasil.

**Keywords:** Neglected Diseases, Bibliometrics, Citation Databases, Doenças Negligenciadas, Bibliometria, Bases de Dados de Citações, Enfermedades Desatendidas, Bibliometría, Bases de Datos de Citas

## Abstract

This study aimed to analyze scientometric profile of publications on neglected tropical diseases (NTDs) in Haiti, 1923-2022. We conducted a scientometric study based on indexed scientific productions related to NTDs in Haiti. The search syntax in the Scopus database was based on key terms for NTDs recognized by the World Health Organization in 2022, focusing on keywords, titles, and abstracts. VOSviewer 1.6.18 was used to create bibliographic networks according to authorship, country of origin, institution, and descriptors. A total of 281 publications were identified, 87.9% (247/281) original articles, with an increase of 45.1% (127/281, annual average of 12.7 publications) in the last decade (2013-2022) compared to the first half of the analyzed period (1923-1975, annual average of 0.21 publications). The authors with the highest number of publications were: Lammie PJ (n = 51), Eberhard ML (n = 29), and Wallace RM (n = 25). A total of 83 institutions participated in the research and the Centers for Disease Control and Prevention (United States) had the highest proportion of affiliations in the coauthorship versus organizations analysis (n = 86), with authors from 35 countries. Coauthorship by country shows publications between institutions in the Americas (n = 13), Europe (n = 9), and Asia (n = 5). The analysis of cooccurrence versus author keywords shows higher occurrence of the terms “dengue” and “rabies”. There is a very limited number of publications on NTDs in Haiti throughout a century of publications, despite the relative increase in recent decades. Such publications are concentrated among foreign authors, with limited national coauthorship.

## Abstract (pt)

O objetivo foi analisar o perfil cienciométrico das publicações sobre doenças tropicais negligenciadas (DTNs) no Haiti de 1923 a 2022. Foi realizado um estudo cienciométrico baseado em produções científicas indexadas relacionadas às DTNs no Haiti. A sintaxe de busca na base de indexação Scopus foi baseada em termos-chave para DTNs reconhecidos pela Organização Mundial da Saúde em 2022, com foco em palavras-chave, títulos e resumos. VOSviewer 1.6.18 foi utilizado para criar redes bibliográficas de acordo com autoria, país de origem, instituição e descritores. Foram identificadas 281 publicações, 87,9% (247/281) artigos originais, com um aumento de 45,1% (127/281, média anual de 12,7 publicações) na última década (2013-2022) face à primeira metade do período analisado (1923-1975, média anual de 0,21 publicações). Os autores com maior número de publicações foram: Lammie PJ (n = 51), Eberhard ML (n = 29) e Wallace RM (n = 25). Participaram da pesquisa 83 instituições, sendo que o Centro de Controle e Prevenção de Doenças (Estados Unidos) apresentou a maior proporção de afiliações na análise de coautoria *versus* organizações (n = 86), com autores de 35 países. Coautoria por país mostra publicações entre instituições das Américas (n = 13), Europa (n = 9) e Ásia (n = 5). A análise de coocorrência *versus* palavras-chave do autor mostra maior ocorrência dos termos “dengue” e “raiva”. Há um número muito limitado de publicações sobre DTNs no Haiti ao longo de um século de publicações, apesar do aumento relativo nas últimas décadas. Essas publicações estão concentradas entre autores estrangeiros, com coautoria nacional limitada.

## Abstract (es)

El objetivo de este estudio fue analizar el perfil cienciométrico de las publicaciones sobre enfermedades tropicales desatendidas (ETD) en Haití en el período de 1923 a 2022. En Haití se realizó un estudio cienciométrico con base en las producciones científicas indexadas relacionadas con las ETD. La búsqueda en la base de indexación de Scopus se basó en términos clave para ETD reconocidos por la Organización Mundial de la Salud en 2022, centrándose en palabras-clave, títulos y resúmenes. Se utilizó VOSviewer 1.6.18 para crear redes bibliográficas según autoría, país de origen, institución y descriptores. Se identificaron 281 publicaciones, un 87,9% (247/281) de artículos originales, con un aumento de un 45,1% (127/281, promedio anual de 12,7 publicaciones) en la última década (2013-2022) en comparación con la primera mitad del período analizado (1923-1975, promedio anual de 0,21 publicaciones). Los autores con mayor número de publicaciones fueron: Lammie PJ (n = 51), Eberhard ML (n = 29) y Wallace RM (n = 25). En la investigación participaron 83 instituciones, y los Centros para el Control y la Prevención de Enfermedades (Estados Unidos) tuvieron la mayor proporción de afiliaciones en el análisis de coautoría *versus* organizaciones (n = 86), con autores de 35 países. La coautoría por país consta de publicaciones entre instituciones de las Américas (n = 13), Europa (n = 9) y Asia (n = 5). El análisis de coocurrencia *versus* de las palabras clave del autor indica una mayor ocurrencia de los términos “dengue” y “rabia”. Hay un número muy limitado de publicaciones sobre ETD en Haití a lo largo de un siglo de publicaciones, a pesar de un aumento relativo en las últimas décadas. Estas publicaciones se concentran entre autores extranjeros, con coautoría nacional limitada.

## Introduction

Neglected tropical diseases (NTDs) are diseases/health conditions that have a significant impact on poorer people and resource-poor communities, with high morbidity and mortality, affecting more than 1 billion people worldwide. They cause serious consequences to physical and mental health and economic impacts ^1^.

The high burden of NTDs in marginalized populations is usually associated with different contexts of vulnerability, such as low levels of education, migration, gender, and black or Indigenous populations ^2^. NTDs are also associated with inadequate access to drinking water, sanitation, basic hygiene, lack of access to healthcare services, and food insecurity ^3^. The incidence of such diseases can reduce cognitive ability and productivity, affect physical health, and limit full human potential and purchasing power ^2^. These aspects perpetuate cycles of vulnerability in a family’s different generations.

The World Health Organization (WHO) has included 21 NTDs in its priority list, which can be grouped according to their causative agents: bacteria (leprosy, trachoma, Buruli ulcer, noma and yaws), viruses (dengue fever, chikungunya, rabies), ectoparasites (scabies, tungiasis, pediculosis, myiasis, cutaneous larvae migrans), protozoa (Chagas disease, leishmaniasis, human African trypanosomiasis), helminths (schistosomiasis, soil-transmitted helminthiases, ancylostomiasis, lymphatic filariasis, onchocerciasis, echinococcosis, dracunculiasis, fascioliasis), fungi (mycetoma, histoplasmosis, cryptococcosis), and snakebite envenoming, recognized as a situation requiring prevention and control measures ^4^.

In Latin America and the Caribbean, about 201 million people (32.1% of the population of 626.2 million) live below the poverty line ^5^, and about 200 million people are affected by NTDs. Regarding prevalence and years of healthy life lost due to disability, soil-transmitted helminthiases and Chagas disease are the most important NTDs in this region, followed by dengue, schistosomiasis, leishmaniasis, trachoma, leprosy, and lymphatic filariasis ^6^.

NTDs in Latin America and the Caribbean are characterized by two main patterns of distribution. First, there is generalized endemicity, as observed for soil-transmitted helminth infections, Chagas disease, and dengue fever ^7^. Second, there is geographically restricted endemicity resulting from planned public health interventions and ecological conditions, such as that observed for onchocerciasis, lymphatic filariasis, and schistosomiasis in parts of the Caribbean, including Haiti ^7^.

In Haiti, the poorest country in the Western Hemisphere, NTDs cause high levels of morbidity and mortality ^8^. Some diseases have been recognized as a public health problem by the Haitian Ministry of Public Health, which has set control program goals by 2030 for a range of NTDs, including lymphatic filariasis, soil-transmitted intestinal helminthiasis, arboviruses, and human rabies, as well as neglected bacterial skin infections (carbuncle) and mansonellosis, which are not included in the list of NTDs made by the WHO ^9^ ^,^ ^10^. In 2018, the Haitian Ministry of Public Health sought to advance the mass drug distribution campaign in 28 priority areas of the country, reaching more than 3 million beneficiaries ^9^. However, for some of NTDs of public health importance, such as cysticercosis, there are no consistent estimates of the burden of disease in Haiti ^6^.

Human rabies is a major health risk in Haiti and it is estimated that up to 130 people die each year from dog-transmitted rabies ^11^. In 2009, the Pan American Health Organization (PAHO) identified the Republic of Haiti as a priority Caribbean country for rabies control. In 2014, it was estimated that 95,000 animal bites occur in Haiti each year. However, only 6,500 bites (6.8%) were reported via the national surveillance program ^12^. Despite the diligent efforts of the national authorities to eliminate this disease, it remains a threat to the population ^13^ ^,^ ^14^ ^,^ ^15^.

Therefore, the context of NTDs in Haiti calls for strong control measures based on scientific evidence. Scientometric surveys are a well-established research method in the information sciences to analyze trends and knowledge development in different fields ^16^ ^,^ ^17^. Given the endemic contexts of Haiti, Latin America and Asia, most publications on NTDs may not reflect the real need to create evidence for the development of control interventions ^6^ ^,^ ^7^. Scientific research in Haiti has been limited and restricted to a few conditions ^7^. From this perspective, scientometrics is relevant to better understand the profile of research on NTDs in the country. This article aims to analyze the scientometric profile of scientific publications on NTDs in Haiti from 1923 to 2022.

## Methods

### Study design

Publications indexed in the Scopus database and accessed via the Federated Academic Community of the Brazilian Coordination for the Improvement of Higher Education Personnel (CAFe-CAPES) (https://www.scopus.com/home.uri) accessed by Federal University of Ceará were analyzed. All types of scientific production on NTDs conducted in Haiti were included, as well as: original articles, reviews, book chapters, conference papers, letters, notes, editorials, and short surveys.

### Study area

Haiti is located in the Caribbean and shares the island of Hispaniola with the Dominican Republic to the east. It has a territorial area of 27,750 km^2^. The territory of the Republic of Haiti includes the western part of Hispaniola and the adjacent islands: Gonâve, Tortue, Ile à Vache, Caimites, Navase, Grande Cayenne, and other islands in the territorial sea. It borders the Dominican Republic to the east, the Atlantic Ocean to the north, and the Caribbean Sea to the south and west ^18^.

The country is divided into 10 departments (equivalent to regions in other countries), 42 districts, 144 municipalities (equivalent to districts, the unit of programmatic implementation for all health programs), 64 neighborhoods and 571 communal (municipal) sections. The geographical departments are North, North-East, North-West, Centre, Artibonite, South, South-East, Nippes, Grand’Anse, and West, with the metropolitan area comprising the urban areas of the six communes of the West department, including the country’s capital, Port-au-Prince ^19^.

The country’s population is estimated to reach 11.9 million in 2021, an increase of 13% in ten years. The population density is 404 inhabitants/km^2^ ^19^. Demographically, the Haitian population is predominantly young and is undergoing an intense and disorganized urbanization process ^20^.

It is the poorest country in the Western Hemisphere, with around a quarter of the population living in extreme poverty, particularly in rural areas. Poverty is reflected in precarious socioeconomic indicators such as high maternal and neonatal mortality, high illiteracy, gender inequality, and low life expectancy ^21^. The country’s location remains conducive to recurrent environmental disasters, with political-institutional instability and increased violence ^22^ ^,^ ^23^. Climate change in recent years also had a significant impact on increasing food insecurity in the country and on the incidence of NTDs. A significant proportion of the population still do not have direct access to electricity, drinking water, basic sanitation, or comprehensive healthcare ^20^ ^,^ ^24^.

### Data source and organization

The database search in June 2023 was based on specific criteria in the advanced search function, using specific author, title, and abstract descriptors, combining specific terms to each NTD (Box 1). The NTDs were selected based on a group of 20 diseases defined by the WHO.

**Box 1:** Neglected tropical diseases (NTDs) and search terms used.

NTDs	SEARCH TERMS
Buruli ulcer	*Buruli ulcer; Mycobacterium ulcerans*
Chagas disease	*Chagas disease; Trypanosoma cruzi*
Chromomycosis	*Chromomycosis; Phialophora; Rhinocladiella; Exophiala; Fonsecaea pedrosoi; Cladophialophora carrionii*
Taeniasis/Cysticercosis	*Cysticercosis; Taeniasis; Taenia solium; Taenia saginata*
Dengue	Dengue; DENV; Flavivírus*
Chikungunya	*Chikungunya fever; Chikungunya virus; CHIKV*
Echinocococosis/Hydatidosis	*Echinococcosis; Echinococcus granulosus; Echinococcus multilocularis*
Fascioliasis	*Fascioliasis; Fasciola gigantica; Fasciola hepatica*
Leishmaniasis	Leishmaniasis; Leishmania donovani; Leishmania chagasi; Leishmania infantum; Leishmania major; Leishmania tropica; Leishmania braziliensis; Leishmania mexicana; Leishmania*
Leprosy	*Leprosy; Mycobacterium leprae*
Elephantiasis/Elephantiasis/Filarial	*Elephantiasis; Filarial; Wuchereria bancrofti; Brugia malayi; Brugia timor*
Mycetoma	*Mycetoma; Nocardia brasiliensis; Nocardia asteroides; Nocardia otitidiscaviarum; Nocardia ninae; Gordonia terrae; Madurella mycetomatis; Fonsecaea pedrosoi; Acremonium falciforme*
Yaws	*Yaws; Treponema pallidum*
Onchocerciasis	*Onchocerciasis; Onchocerca volvulus*
Rabies	*Rabies; Rabies virus*
Schistosomiasis	*Schistosomiasis; Schistosoma haematobium; Schistosoma guineensis; Schistosoma intercalatum; Schistosomiasis japonica; Schistosoma mekongi; Schistosomiasis mansoni*
Trachoma	*Trachoma; Chlamydia trachomatis*
Ascariasis	*Ascariasis; Ascaris lumbricoides; Ascaris suum*
Trichuriasis	*Trichuriasis; Trichocephalus; Trichuris trichiura*
Ancylostomiasis	*Ancylostomiasis; Ancylostoma caninum; Necator americanus*
Dracunculiasis	*Dracunculiasis; Dracunculus medinensis*
Clonorchiasis	*Clonorchiasis; Clonorchis sinensis*
Paragonimiasis	Paragonimiasis; Paragonimus*
Opisthorchiasis	*Opisthorchiasis; Opisthorchis viverrine; Opisthorchis felineus*
Trypanosomiasis, African	*Trypanosomiasis; African; Trypanosoma brucei gambiense; Trypanosoma brucei rhodesiense*
Snake bites	*Snake bites*
Histoplasmosis	*Histoplasmosis; Histoplasma capsulatum*
Coccidioidomycosis	*Coccidioidomycosis; Coccidioides immitis; Coccidioides posadasii*
Chromoblastomycosis	*Chromoblastomycosis; Fonsecaea pedrosoi; Phialophora verrucosa; Cladophialophora carrionii; Rhinocladiella aquaspersa*
Paracoccidioidomycosis	*Paracoccidioidomycosis; Paracoccidioides brasiliensis*
Sporotrichosis	*Sporotrichosis; Sporothrix schenckii*
Cryptococcosis	*Cryptococcosis; Cryptococcus neoformans; Cryptococcus gattii*
Scabies	*Scabies; Sarcoptes scabiei*
Tungiasis	*Tungiasis; Tunga penetrans*
Cutaneous larva migrans	*Cutaneous larva migrans; Visceral larva migrans; Ancylostoma caninum; Ancylostoma brasiliensis; Strongyloides stercoralis*
Head lice infestations	*Lice infestations; Pediculus humanus capitis; Body lice; Phtiriase*
Myiasis	*Myiasis; Cochliomyia hominivorax; Oestrus ovis; Wohlfahrtia magnifica; Chrysomya bezziana; Hypoderma bovis; Hypoderma lineatum; Cordylobia anthropophaga; Hypoderma tarandi; Calliphora vicina; Musca nebulo; Musca domestica; Lucilia sericata*

All publications from January 1923 to December 2022 were included. All authors (with their country of origin and institutional affiliation) included in the scientific publications were considered and the health descriptors in each publication were extracted and analyzed. The number and proportion of identified scientific publications on NTDs over time was assessed.

The analysis of the data was conducted using the scientometric visualization software VOSviewer 1.6.16 (https://www.vosviewer.com/), based on the structuring of the bibliographic networks and the specifics of the references associated with each record, as well as the author’s descriptor data and the arrangement of the most frequent terms in the publications.

The descriptive analysis covered the entire period (1923-2022), with the construction of graphs to characterize the evolution of scientific production over time. Subsets of data relating to the number and proportion of publications on the different NTDs in Haiti were also analyzed, identifying such diseases with the highest proportion of scientific publications. Tables were created to show the ten most common types of scientific products, as well as authors, descriptors, affiliations, and countries.

### Analysis of the bibliometric profile

Graphical representations were made of the relationships (maps) between authors, countries, institutions and descriptors (nodes), the strength between these relationships (thickness of arcs), and the number of their total contributions (node size). The VOSviewer “Thesaurus” tool was used to consolidate the terms. The parameters were defined with a maximum of 25 and a minimum of 2 for the scientometric identification items for each unit of analysis: (1) coauthorship by author, (2) coauthorship by country, (3) coauthorship by organization, and (4) cooccurrence of author keywords, linked to the network of the bibliographic production relationship and aggregated over the entire study period.

### Ethical aspects

This study was based on publications on NTDs indexed in the Scopus database, which is free and open access, via CAFe-CAPES, to which the Federal University of Ceará has access. According to Brazilian guidelines there is no need to submit the study to a research ethics committee.

## Results

**Figure 1:**
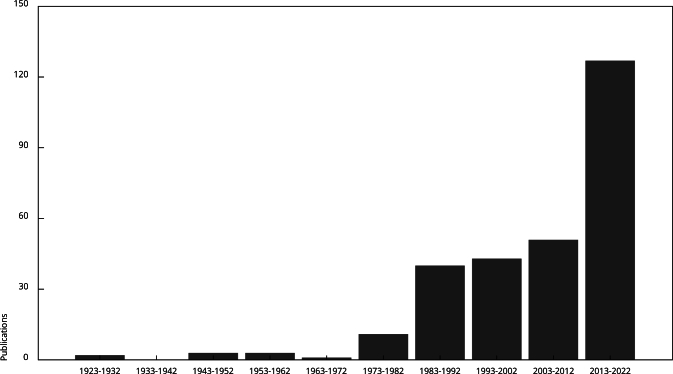
Publications on neglected tropical diseases (NTDs) in Haiti, 1923-2022 (n = 281).

A total of 281 publications were identified, with an increase of 66.8% (127/281, annual average of 12.7 publications) in the last decade (2013-2022), compared to the first five decades (1923-1972, annual average of 0.19 publications) (Figure 1).

Original articles were the most common type of publication, accounting for 87.9% (n = 247) of all publications, followed by systematic reviews (8.7%; n = 22) (Table 1).

**Table 1:** Number and percentage of articles on neglected tropical diseases, according to the type of document, and top 10 authors, countries, institutions, and keywords, 1923-2022.

Profile	n	%
Total	281	100.0
Type of document		
Article	247	87.9
Review	22	7.8
Book chapter	3	1.0
Conference paper	2	0.7
Letter	2	0.7
Note	2	0.7
Editorial	1	0.4
Erratum	1	0.4
Short survey	1	0.4
Authors		
Lammie PJ	51	18.1
Eberhard ML	29	10.3
Wallace RM	25	8.9
Addis DG	22	7.8
Crowdis K	17	6.0
Blanton JD	15	5.3
Raccurt CP	15	5.3
Etheart MD	14	5.0
Millien MF	14	5.0
Streit TG	12	4.3
Countries		
United States	195	69.4
Haiti	83	29.5
France	23	8.2
Brazil	16	5.7
United Kingdom	16	5.7
Canada	8	2.8
Chile	6	2.1
Italy	6	2.1
Germany	5	1.8
India	4	1.4
Institutions		
Centers for Disease Control and Prevention, Atlanta, Georgia, United States	86	30.6
Haitian Ministry of Public Health, Port-au-Prince, Haiti	22	7.8
University of Florida, Gainesville, Florida, United States	18	6.4
Centers for Disease Control and Prevention, Haiti Country Office, Port-au-Prince, Haiti	15	5.3
Haitian Ministry of Agriculture, Rural Development and Natural Resources, Port-au-Prince, Haiti	15	5.3
Christian Veterinary Mission	14	5.0
Sainte Croix Hospital, Leogane, Haiti	13	4.6
University of Notre Dame, Notre Dame, Indiana, United States	8	2.8
Emory University, Atlanta, Georgia, United States	8	2.8
Tulane University Medical Center, New Orleans, Louisiana, United States	8	2.8
Keywords		
Dengue	13	4.6
Rabies	12	4.3
Chikungunya	9	3.2
Lymphatic filariasis	9	3.2
*Aedes aegypti*	5	1.8
Caribbean	7	2.5
Zoonotic diseases	5	1.8
*Wuchereria broncrofti*	5	1.8
Leprosy	4	1.4
Epidemiology	5	1.8

The authors with the highest number of publications were: Lammie PJ (n = 51), Eberhard ML (n = 29), Wallace RM (n = 25), and Addis DG (n = 22). Authors came from 65 countries, most from the United States (n = 195), followed by Haiti (n = 83) and France (n = 23) (Table 1).

A total of 83 institutions were associated with the productions (coauthors versus organizations), with the Centers for Disease Control and Prevention (CDC; United States) having the largest representation (n = 86), followed by the Haitian Ministry of Public Health (n = 22), the University of Florida (United States) (n = 18), the CDC Haiti (n = 15), and the Haitian Ministry of Agriculture, Rural Development and Natural Resources (n = 15) (Table 1).

Of the total number of publications, 65.8% (n = 185) were developed by institutions in Haiti, with most studies conducted in the West Department (n = 75; 40.5%). The most published NTDs were: filariasis (78; 27.8%), dengue fever (40; 14.2%) and rabies (33; 11.7%). Overall, 328 descriptors were identified in these publications and the term “dengue” (n = 13) was the most frequently used keyword, followed by “rabies” (n = 12) (Table 1).

**Figure 2:**
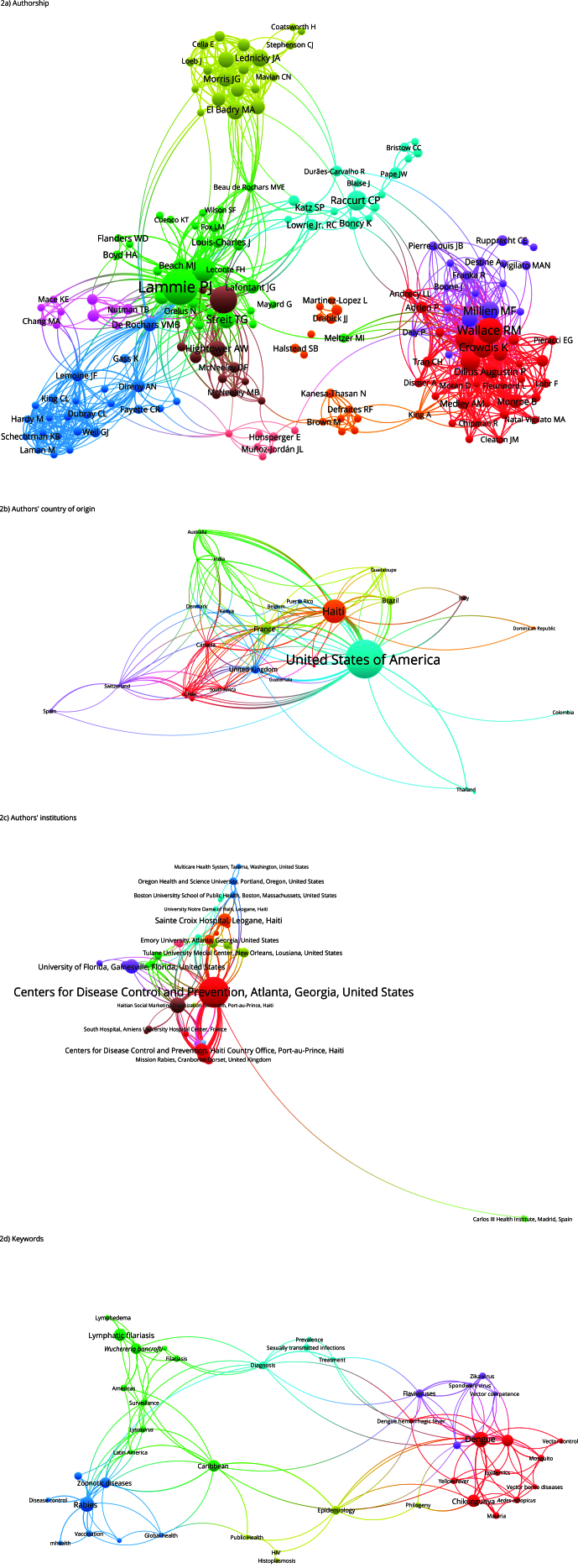
Networks of scientific productions on neglected tropical diseases. Haiti, 1923-2022.

The coauthorship to author ratio identified 1,113 authors, of which 219 were selected for analysis, organized into ten clusters, with cluster 1 having a total of 31 authors, followed by cluster 2 with 29 authors, and cluster 3 with 24 authors. The authors with the most connections were Lammie PJ, Wallace RM and Crowdis K; Lammie PJ also stands out as the author with the most citations and connections with Addiss DG and Eberhard ML (Figure 2a).

The relationship between coauthorship and country indicates publications in institutions in the Americas (n = 13), Europe (n = 9), Asia (n = 5), Africa (n = 4), and Oceania (n = 3). The main relationship was the United States-Haiti interface. The analysis identified 9 clusters, with 8 countries in the main cluster, 6 in the second cluster, and 5 in the third cluster (Figure 2b).

It showed relationships with an established pattern between coauthors and organizations, identifying 600 institutions, of which 75 were selected for scientometric analysis. The main relationship identified was between the CDC and the Department of Agriculture, Rural Development and Natural Resources. This analysis identified 12 clusters, with the main cluster containing 11 institutions, followed by a cluster with 9 institutions, and the third cluster with 7 institutions (Figure 2c).

In the analysis of the cooccurrence relationship with author keywords, 328 keywords were identified, of which 50 were selected for analysis, organized in 6 clusters. The main cluster had 11 items, followed by cluster 2 with 9 items, and cluster 3 with 8 items. The most prominent terms were “dengue”, “rabies”, “chikungunya”, and “*Aedes aegypti*”, with direct relationships between terms related to arboviruses (Figure 2d).

## Discussion

This unprecedented study shows that there are only a limited number of studies on NTDs in Haiti, despite the high social vulnerability associated with this group of diseases. Furthermore, most publications related to such studies have been produced by foreign researchers. Despite a century of publications, only 281 publications on NTDs were identified, demonstrating the lack of research in the country, which reduces the possibility of human and social development based on the diseases recognition to plan and implement evidence-based control measures ^25^.

The increase of almost 70% in the annual average number of publications since 1970 is probably related to the process of indexing article abstracts in the Scopus database, indicating increasing mobilization over time. Despite the observed upward trend, critical gaps remain and may reaffirm the nature of NTDs ^26^, not only because of scientific failures but also because of failures of the public policy in national health systems ^27^.

The involvement of 75 institutions with effective participation in publications by authors from 35 countries highlights the global nature of NTD research. There is a clear predominance of researchers from the United States, indicating the country’s strong involvement in Haitian research from different perspectives related to NTDs. This is evidenced by their identification among the top ten most productive authors such as Patrick. J. Lammie of the Cell Biology Branch of the CDC. Such researchers establish broad and significant links globally, in diverse collaborations, to conduct studies in institutions that have potential access to financial resources to support research ^28^. Relationships between coauthors and organizations reveal a pattern between institutions over the period 1923-2022.

The U.S. influence on Haiti dates back long before the World War I ^29^. Haiti was the second country in the Americas to achieve their independence, but the United States did not recognize it until more than half a century later, fearing that achieving their independence via a slave revolution could cause internal instability in the country. As a result, its process of domination has continued unabated into the 20th and 21st centuries ^30^. The U.S. dominance in the context of academic research in Haiti may reflect its strong political, social, and economic influence on the scientific field, as well as its strategic sphere of influence in the country ^31^.

The analysis of scientific production also highlighted the participation of more active institutions, with leadership in NTD research, such as the CDC (United States/Haiti) and the Haitian Ministry of Public Health ^32^. The CDC is a U.S. institution that has been present in Haiti since 2002. Its work has enabled Haiti’s national surveillance network to expand from 51 sites in 2010 to 652 sites in 2018, covering more than 60% of the country’s health facilities. CDC has supported the Haitian Ministry of Public Health to strengthen public health interventions in the country ^14^ ^,^ ^33^. The strong collaboration between Haiti and countries such as the United States, France, Brazil, and the United Kingdom also demonstrates the importance of international cooperation in NTDs control and global health research ^4^.

Keyword cooccurrence analysis highlights terms such as “dengue”, “rabies”, “chikungunya”, and “lymphatic filariasis”, reflecting the focus of research during the analyzed period. Some NTDs defined by the WHO are recognized as a public health problem in Haiti, with a list of around ten diseases, many of which are already subject of control programs under the country’s health plan (*Plan Directeur de Santé*; PDS) up to 2030 ^4^, such as lymphatic filariasis, rabies, and helminthiases ^34^. Although all these diseases are considered prevalent in Haiti, limitations in research development and knowledge generation remain a critical constraint for their epidemiological control ^9^.

Arboviruses and human rabies have been the most common in published studies. In fact, up to 130 people die each year in Haiti from dog-transmitted rabies ^13^ ^,^ ^35^ ^,^ ^36^. Despite disease control measures taken by the national authorities, rabies remains a public health problem in the country ^14^.

The social determinants present in Haiti create the potential for a high incidence of several NTDs. The population is socially vulnerable (59% of citizens live below the poverty line) and programmatically vulnerable, with limited access to diagnosis and treatment in the national health system. There is a significant under-five mortality rate of 76 per 1,000 live births, and only half of the population has access to piped water ^37^ ^,^ ^38^.

The Haiti population potentially lives in areas at risk of filariasis and helminthiases. The development of health interventions, including NTDs control plans, is organized at the commune level ^39^. However, this process is complex, as approximately 60% of the population lives in rural areas and 40% in four large urban areas, the largest of which is the capital, Port-au-Prince (population 2.5 million) ^14^.

The country has faced major challenges in advancing its economic and social development ^8^. Examples of political and institutional crises include hurricanes and a 7.2 magnitude earthquake (August 2021) that hit the southern region of the country (where approximately 1.6 million people live), followed by a significant cholera outbreak in the region ^34^. In addition, political instability negatively affects, among other things, the implementation of the national health policy, contributing to the emergence and persistence of NTDs ^40^. Amid this scenario, the country has also faced high population growth, with accelerated and disorganized urbanization, which has also influenced the growth of cities, generally associated with extreme poverty, unemployment, inadequate housing, subnormal agglomerations with a strong presence of vector-borne diseases, environmental degradation, and pollution ^41^.

Knowledge production keeps facing challenges related to epistemological, geopolitical, economic and social development issues, and that the localization of such diseases is entirely linked to colonial and capitalist dynamics, highlighting the fact that policies are still conceived in an asymmetrical way between peoples and nations. This study indirectly reveals the historical vulnerability of the Haitian population in knowledge production and puts into perspective the need for a global health agenda that understands the dimension of neglected bodies and populations, going beyond the health perspective and considering analyses that integrate political, social, and economic contexts ^42^.

As a result, most publications in Haiti continue to be published by researchers from high-income countries. However, there are established links between Haitian authors and institutions in other Latin American countries, Europe, Oceania, and Asia. The under-representation of low- and middle-income countries in scientific production implies an understanding of endemic diseases that may not reflect local realities. The limited number of publications limits attempts to develop global strategies based on consistent evidence that are effective. In addition, there are scientific, market, and public health failures to achieve disease control in these places ^43^.

The analyzed productions were developed in different contexts in the ten departments of Haiti, with the West Department standing out as the one where lymphatic filariasis is the most recognized disease in scientific research in the region ^44^ ^,^ ^45^. This may be related to the production of research targeting marginalized populations in filariasis-endemic contexts ^45^ ^,^ ^46^, and highlights the need for greater recognition of data from other regions to support and inform decision making in surveillance programes ^35^ ^,^ ^46^. In addition to filariasis, diseases such as dengue and rabies are also among the most studied NTDs.

The Scopus database was chosen as the reference for this study because it has the largest collection of publications on endemic NTDs in Haiti. This database considers the development of research in other Latin American countries, in which dengue is highly represented, followed by leishmaniasis, trachoma, leprosy, lymphatic filariasis, Chagas disease, and schistosomiasis ^47^. The fact that the Scopus database takes a broader perspective in this context made it possible to identify NTDs with a greater number of publications, including those that are well-established public health problems for the country ^6^.

The presence of the term “NTD” as a descriptor may be related to the common and established use of this nomenclature for this group of diseases. In addition, the availability of the term as a scientific descriptor in the main databases for indexing publications, such as Embase, Cochrane Library, DARE, MEDLINE, and PubMed Health, reflects the performance of each database. A combination of databases showed an increase in sensitivity, with Embase being superior to Cochrane Library ^48^.

The limitations of this study are related to the scope of the data collection and the indexing process of the Scopus indexing database. Despite the broad coverage, with indexing of a significant number of articles and scientific journals and other websites publishing research, note that no single database was able to identify all relevant systematic reviews published on NTDs. The limited amount of data published in Haiti may contribute to a lack of understanding of the incidence and social determinants of endemic diseases in the country, thus limiting evidence for the adoption of more effective control measures.

## Conclusion

There has been a limited number of scientific publications on NTDs in Haiti over the past 100 years, despite a relative increase in the last decade. Such publications are concentrated among foreign authors, with limited national coauthorship, and do not cover all endemic areas or even all the most prevalent diseases in the country.

The persistence of NTDs as a public health problem in Haiti highlights the need for greater investment in science, technology, and innovation. Our study reinforces the need to expand local knowledge production in specific contexts and regions, with a strong focus on global articulation. To this end, national agendas need to increase investment and effective international collaboration for the sustainability of actions in national institutions that support the national response to NTDs.

## References

[1] World Health Organization Control of neglected tropical diseases..

[2] Molyneux D Maladies tropicales négligées..

[3] The Lancet (2022). Neglected tropical diseases: ending the neglect of
populations.. Lancet.

[4] World Health Organization Neglected tropical diseases..

[5] Comisión Económica para América Latina y el Caribe Panorama social de América Latina y el Caribe..

[6] Hotez PJ, Bottazzi ME, Franco-Paredes C, Ault SK, Periago MR (2008). The neglected tropical diseases of Latin America and the
Caribbean a review of disease burden and distribution and a roadmap for
control and elimination. PLoS Negl Trop Dis.

[7] Fontecha G, Sánchez A, Ortiz B (2021). Publication trends in neglected tropical diseases of Latin
America and the Caribbean a bibliometric analysis. Pathogens.

[8] Alsan MM, Westerhaus M, Herce M, Nakashima K, Farmer PE (2011). Poverty, global health, and infectious disease lessons from Haiti
and Rwanda. Infect Dis Clin North Am.

[9] Raccurt CP, Boncy J, Jean-Baptiste RMA, Honoré R, Andrecy LL, Dély P (2018). Update of knowledge on neglected diseases in Haiti
mansonelliasis, tungiasis, leprosy, and anthrax. Bull Soc Pathol Exot.

[10] Hast MA, Javel A, Denis E, Barbre K, Rigodon J, Robinson K (2022). Positive-case follow up for lymphatic filariasis after a
transmission assessment survey in Haiti. PLoS Negl Trop Dis.

[11] Wallace RM, Etheart MD, Doty J, Monroe B, Crowdis K, Dilius Augustin P (2016). Dog-mediated human rabies death, Haiti, 2016. Emerg Infect Dis.

[12] Osinubi MOV, Fenelon N, Dyer JL, Franka R, Etheart M, Ali A (2018). Meeting the urgent need for rabies education in
Haiti. Zoonoses Public Health.

[13] Hampson K, Coudeville L, Lembo T, Sambo M, Kieffer A, Attlan M (2015). Estimating the global burden of endemic canine
rabies. PLoS Negl Trop Dis.

[14] Lemoine JF, Desormeaux AM, Monestime F, Fayette CR, Desir L, Direny AN (2016). Controlling neglected tropical diseases (NTDs) in Haiti
implementation strategies and evidence of their success. PLoS Negl Trop Dis.

[15] Millien MF, Pierre-Louis JB, Wallace R, Caldas E, Rwangabgoba JM, Poncelet JL (2015). Control of dog mediated human rabies in Haiti no time to
spare. PLoS Negl Trop Dis.

[16] Blake RM, Adams J Global research report ? neglected tropical diseases..

[17] Malecela MN (2019). Reflections on the decade of the neglected tropical
diseases. Int Health.

[18] République d'Haïti 1987 Constitution de la République d'Haïti..

[19] Institut Haïtien de Statistique et d'Informatique Les comptes economiques en 2022..

[20] United Nations Children's Fund Country office annual report 2019: Haiti..

[21] The World Bank Haiti: data..

[22] Charles R, Oliveira RC, Coltri PP, São José RV (2020). The vulnerability of Haiti in front of climate
variability. Revista de Geografia.

[23] Programme des Nations Unies pour l'Environnement; Ministere de
l'Environnement d'Haiti; l'Université Quisqueya État et perspectives de l'environnement. GEO Haïti 2010..

[24] World Food Programme Haiti: annual country report 2022..

[25] Maciel AMS, Ramos AN, Ferreira AF, Almeida NMGS, Gomes VS, Gómez DVF (2022). Scientometric analysis of research on trachoma in Brazil,
2000-2020.. Rev Saúde Pública.

[26] Bai J, Li W, Huang YM, Guo Y (2016). Bibliometric study of research and development for neglected
diseases in the BRICS. Infect Dis Poverty.

[27] Mahoney RT, Morel CM (2006). A Global Health Innovation System (GHIS). Innovation Strategy Today.

[28] González-Alcaide G, Salinas A, Ramos JM (2018). Scientometrics analysis of research activity and collaboration
patterns in Chagas cardiomyopathy. PLoS Negl Trop Dis.

[29] Coggiola O (2016). História do capitalismo: das origens até a Primeira Guerra
Mundial.

[30] Andrade EO (2016). A primeira ocupação militar dos EUA no Haiti e as origens do
totalitarismo haitiano. Revista Eletrônica da ANPHLAC.

[31] Bissindé CA (2023). The relations between Haiti and the United States dependence and
hegemony. Hoplos.

[32] Elbadry MA, Al-Khedery B, Tagliamonte MS, Yowell CA, Raccurt CP, Existe A (2015). High prevalence of asymptomatic malaria infections a
cross-sectional study in rural areas in six departments in
Haiti. Malar J.

[33] Centers for Disease Control and Prevention CDC in Haiti..

[34] Oscar R, Lemoine JF, Direny AN, Desir L, Beau de Rochars VEM, Poirier MJP (2014). Haiti National Program for the Elimination of Lymphatic
Filariasis: a model of success in the face of adversity.. PLoS Negl Trop Dis.

[35] Carvalho MF, Vigilato MAN, Pompei JA, Rocha F, Vokaty A, Molina-Flores B (2018). Rabies in the Americas 1998-2014. PLoS Negl Trop Dis.

[36] Ma X, Blanton JD, Millien MF, Medley AM, Etheart MD, Fénelon N (2020). Quantifying the risk of rabies in biting dogs in
Haiti. Sci Rep.

[37] The World Bank Haiti..

[38] World Health Organization The Global Health Observatory. Haiti..

[39] Losonczy LI, Barnes SL, Liu S, Williams SR, McCurdy MT, Lemos V (2019). Critical care capacity in Haiti a nationwide cross-sectional
survey. PLoS One.

[40] Biermann F, Hickmann T, Sénit CA, Beisheim M, Bernstein S, Chasek P (2022). Scientific evidence on the political impact of the Sustainable
Development Goals. Nat Sustain.

[41] Mahtta R, Fragkias M, Güneralp B, Mahendra A, Reba M, Wentz EA (2022). Urban land expansion the role of population and economic growth
for 300+ cities. NPJ Urban Sustain.

[42] Oliveira RG (2018). Meanings of neglected diseases in the global health agenda the
place of populations and territories. Ciênc Saúde Colet.

[43] Khulbe Y, Chandani Y, Kamaraj B, Agrawal V (2023). Under-representation of low-income countries in the literature -
targeting the bummock of neglected tropical diseases. Trop Doct.

[44] Oliveira M, Castro E, Pisco Pacheco H, Frederico A, Candeias R, Calvo I (2005). TROP7 lesions kystiques musculaires Ne pas oublier
l'echinococcose granulosus primitive. J Radiol (Paris).

[45] Drexler N, Washington CH, Lovegrove M, Grady C, Milord MD, Streit T (2012). Secondary mapping of lymphatic filariasis in Haiti definition of
transmission foci in low-prevalence settings. PLoS Negl Trop Dis.

[46] Champetier de Ribes G, Fline M, Désormeaux AM, Eyma E, Montagut P, Champagne C (2005). Helminthoses intestinales en milieu scolaire en Haïti en
2002.. Bull Soc Pathol Exot.

[47] Ferreira AF, Heukelbach J, Costa CHN, Souza EA, Maciel AMS, Correia D (2023). Scientometric review of research on neglected tropical diseases a
31-year perspective from the Journal of the Brazilian Society of Tropical
Medicine. Rev Soc Bras Med Trop.

[48] Zhang D, Song Q, Zheng Q (2017). Optimizing literature search in systematic reviews is MEDLINE
sufficient for identifying effect studies on corneal properties and
glaucoma?. Arq Bras Oftalmol.

